# Deductive machine learning models for product identification†

**DOI:** 10.1039/d3sc04909d

**Published:** 2024-07-01

**Authors:** Tianfan Jin, Qiyuan Zhao, Andrew B. Schofield, Brett M. Savoie

**Affiliations:** a Department of Chemical Engineering, Purdue University West Lafayette USA bsavoie@purdue.edu

## Abstract

Deductive solution strategies are required in prediction scenarios that are under determined, when contradictory information is available, or more generally wherever one-to-many non-functional mappings occur. In contrast, most contemporary machine learning (ML) in the chemical sciences is inductive learning from example, with a fixed set of features. Chemical workflows are replete with situations requiring deduction, including many aspects of lab automation and spectral interpretation. Here, a general strategy is described for designing and training machine learning models capable of deduction that consists of combining individual inductive models into a larger deductive network. The training and testing of these models is demonstrated on the task of deducing reaction products from a mixture of spectral sources. The resulting models can distinguish between intended and unintended reaction outcomes and identify starting material based on a mixture of spectral sources. The models also perform well on tasks that they were not directly trained on, like performing structural inference using real rather than simulated spectral inputs, predicting minor products from named organic chemistry reactions, identifying reagents and isomers as plausible impurities, and handling missing or conflicting information. A new dataset of 1 124 043 simulated spectra that were generated to train these models is also distributed with this work. These findings demonstrate that deductive bottlenecks for chemical problems are not fundamentally insuperable for ML models.

## Abstract 2

Machine learning models are developed that emulate the H deductive chemical reasoning processes associated with product identification from analytical spectra.

## Graphical abstract

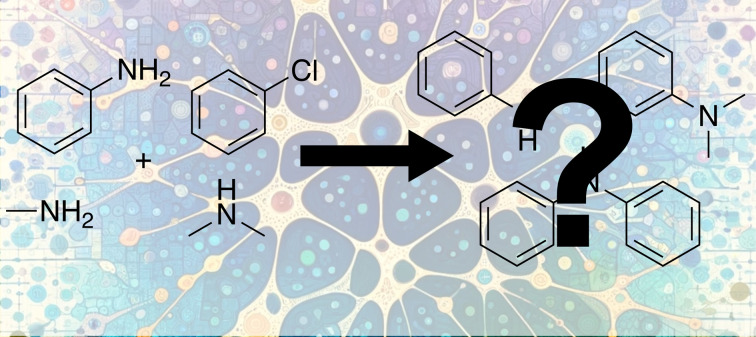

Product identification is a central task in every reaction development workflow.^1–5^ There is no standardized solution to this problem, with practices ranging from separation and crystallization for unequivocal identification, to using a mixture of analytical information sources (*e.g.*, mass spectrometry (MS), nuclear magnetic resonance (NMR), infrared spectroscopy (IR), *etc.*) and general reactivity knowledge to distinguish between plausible products. The lack of standardization reflects that product identification is typically underdetermined by simple knowledge of the reactants and conditions. For example, a new reaction may yield a complex product mixture that requires several iterations of characterization and interpretation to fully identify, and even putatively established reactions can yield unexpected products if a hot-plate fails or a starting material has an impurity. Underdetermination also occurs because most analytical characterizations only provide partial or indirect structural information, and a particular analytical method may yield decisive information for identifying one product but not another.^6–9^ For these reasons, the state-of-the-art for general product identification remains manual expert interpretation of multiple information sources.

Product identification is a member of a larger group of deduction problems that are common in the chemical sciences (Fig. 1A). In deductive scenarios, external information is used to restrict the potential solution space when making a prediction. Deduction is required for underdetermined problems or when there is a mixture of competing information sources. In contrast, most machine learning (ML) in chemistry is inductive, learning from example, with a fixed set of input features.^10–13^ In the case of product identification, deduction takes the form of using established reactivity relationships to narrow the solution space to a small number of potential products that can then be inductively distinguished using one or more analytical spectra. More generally, deduction is needed whenever a non-functional one-to-many relationship exists between input features and prediction targets. In the context of ML, this distinction is critical, because regardless of their complexity, neural networks are incapable of circumventing the information limitations posed by non-functional mappings.

**Fig. 1:**
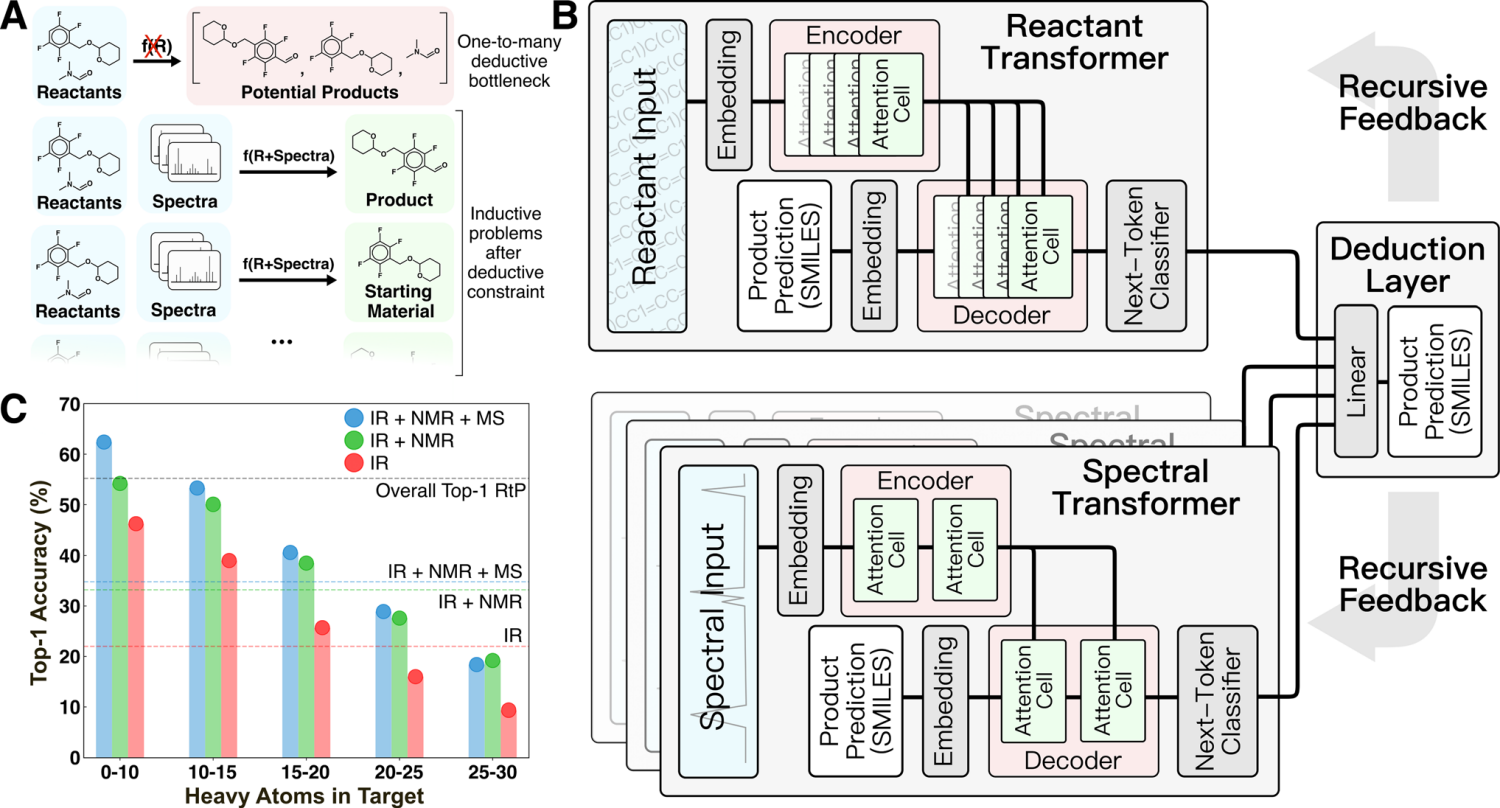
Overview of deductive architecture and bottleneck for product identification. (A) Illustration of the general non-functional one-to-many relationship between reactant information and some potential species that can be found as intended and unintended products. (B) Deductive super-network consisting of a reactant to product (RtP) transformer and one or more spectrum to structure (StS) transformers combined by a terminal linear layer. The model predicts product SMILES in probabilistic token-by-token fashion. (C) Top-1 accuracy of StS models in predicting structures from the testing set with an increasing number of heavy atoms. The dotted lines indicate the overall top-1 accuracy of each model on the whole testing set.

The motivation for the current study was to develop a ML-framework capable of emulating expert deduction to perform product identification based on a flexible mixture of spectral input sources. We hypothesized that deduction would be an emergent property of a super-network composed of individual task-specific inductive neural networks and a method of decomposing the prediction task into subproblems that allows each subnetwork to exercise its competence (Fig. 1B). This idea was directly motivated by the manual analog of interpreting individual spectra to obtain derived information (*e.g.*, identifying the presence of certain functional groups from IR or a probable chemical formula from MS) then forming structural hypotheses from comparisons of this derived information.

Here, we experimented with combining up to four task-specific transformers for ingesting reactant/reagent information and IR, ^1^H-NMR, and electron-ionization (EI) MS spectra, respectively. The overall architecture inputs consist of reactant/reagent graph(s) supplied as simplified molecular-input line-entry system (SMILES)^14^ strings and one or more analytical spectra associated with an unidentified target. These inputs are used to probabilistically decode the product SMILES (*i.e.*, its graph) as an output in recursive token-by-token fashion. This recursion is achieved by supplying the partially decoded product SMILES as an additional input to all transformers until encountering an end token. Each task-specific transformer provides a probabilistic prediction of the next token in the product that informs a final linear deduction layer (see Methods).

This architecture provides two sources of deductive coupling between the transformers. The first is the straightforward probability reweighting that happens in the final linear deduction layer, which provides the opportunity for one or more of the transformers to form a consensus over the other transformer(s). The second is through the recursive token-by-token decoding by which the product prediction is made. Because the partially decoded product string is used as an input to each transformer during inference, it is possible for control to shift between transformers for different portions of the decoding (*e.g.*, one may dominate the scaffold, while another dominates predictions of certain functional groups). In this way, the transformers can dynamically provide deductive constraints on each other during different portions of the decoding. The differing inputs for each transformer and their coupling through the recursive decoding distinguishes this architecture from a simpler ensemble. Recently, similar architectures based on the idea of “Mixture of Experts” have become popular in the large language model community,^15^ but the multi-modal (spectra + graph) input to product graph architecture demonstrated here remains the first of its kind.

The deduction models were trained and tested on 299 658 reactions taken from the Lowe patent dataset after filtering (see Methods).^16,17^ Artificial EI-MS, ^1^H-NMR, and IR spectra were generated for all products, reactants, and reagents due to the unavailability of suitable experimental training data for this task. To turn this into a deductive product identification task, the dataset was augmented with null reactions that corresponded to obtaining starting material from the reaction instead of the expected product. The final dataset consisted of 299 658 real reactions and 146 672 null reactions, that were split using a 80 : 10 : 10 training, validation, testing distribution while ensuring that there were no prediction targets shared between the splits. All accuracies are reported for the testing set.

## Results

### Baseline models suffer from deductive limitations

Prediction baselines for this task were set by training analogous transformer models on the reactant-to-product (RtP) and spectrum-to-structure (StS) prediction tasks (Fig. 1C). The RtP model exhibits an obvious deductive bottleneck in this task, since a given reactant can map to either the expected product or starting material(s). The RtP model was trained only to predict the expected products, because attempts to train with null reactions in the training data led to confusion due to the one-to-many relationship between inputs and targets. Thus, the RtP model serves as a baseline for a model that always predicts the expected product. The RtP model's top-1 accuracy of ∼55% reflects a combined top-1 accuracy of ∼0.6% on null reactions and ∼84.5% on real reactions in the testing set. The latter result is comparable to the state-of-the-art RtP models, which can reach top-1 accuracies of 88.8% when only tested on major product prediction.^18,19^ Several StS models were trained with different combinations of spectral transformers (IR, IR + NMR, and IR + NMR + MS models in Fig. 1B). The StS models exhibit lower overall performance than the RtP model, with a top-1 accuracy of ∼35% for the best model (IR + NMR + MS). The accuracies monotonically increase with the number of spectral sources used in the prediction and monotonically decrease with the molecular size of the prediction target. Although the deductive bottleneck is less obvious, it is qualitatively expected that spectral uniqueness decreases with molecular size (*e.g.*, the structural isomers of large molecules often cannot be distinguished by these spectra). These accuracies favorably compare with recently published StS models that also exhibit relatively low performance for large molecules. For instance, Alberts *et al.*^20^ reported 17% top-1 and 33.6% top-5 accuracy for predicting molecular structure from IR only. Another case study focused on using MS to predict molecular fingerprints, rather than the molecular structure, and reported poor results on an out-of-distribution testing set with 27.8% top-1 and 42.5% top-5 accuracies.^21^ Notably, groups have reported StS accuracies that significantly improve when the molecular formula is supplied to the model in addition to the spectra. For example, Huang *et al.* reported an ^1^H-NMR + formula to structure model with 47.4% top-1 and 85.3% top-10 accuracies.^7^ Although it has not been identified as such, supplying the formula is an elementary deductive constraint.

**Fig. 2:**
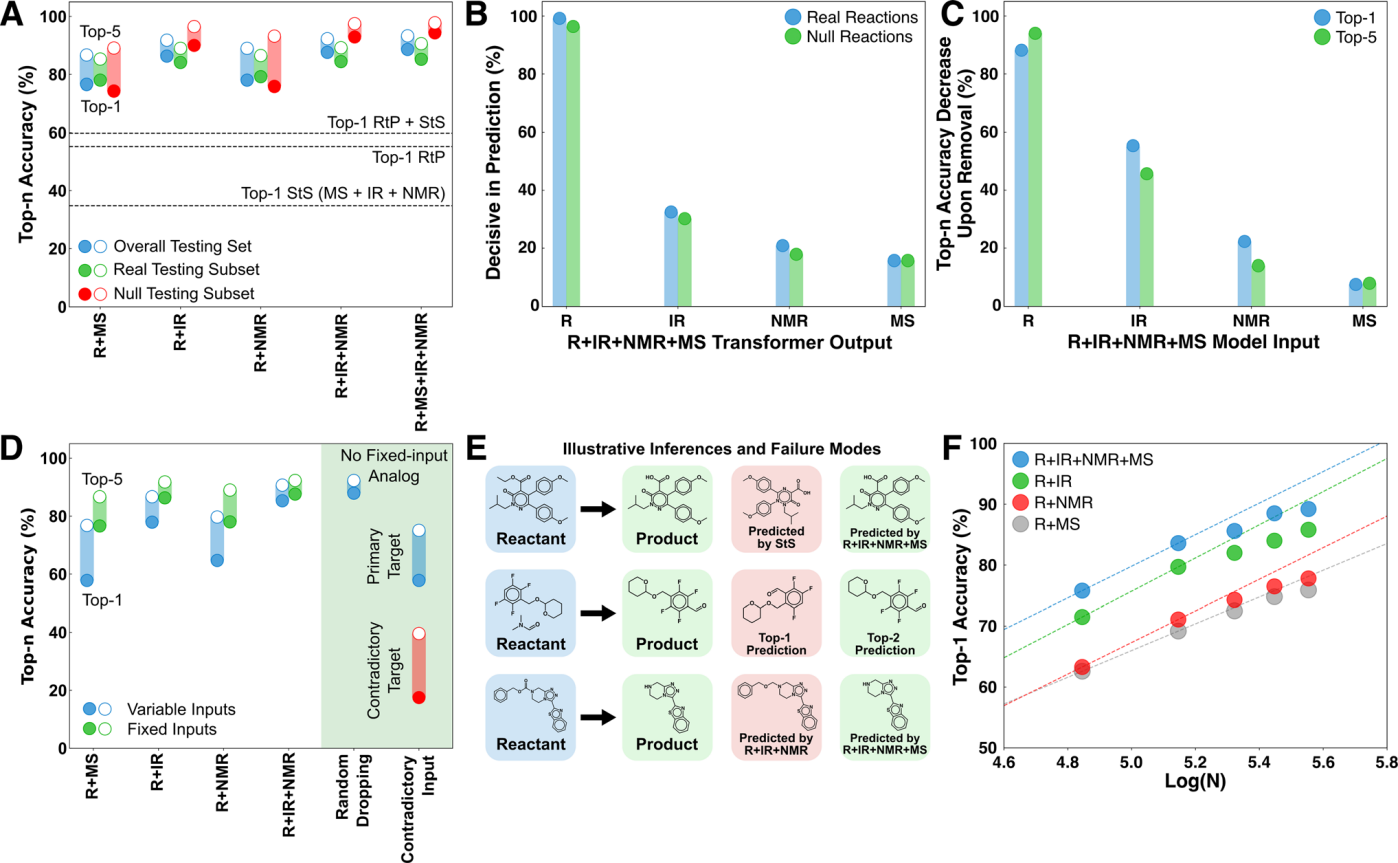
Overview of deductive performance in product identification tasks. (A) Comparison of several reactant + spectrum deductive models with RtP and StS models. The RtP + StS result corresponds to the accuracy obtained by combining the correct predictions from both models. Top-*n* accuracy metrics based on ensembles of independently trained models were within 0.5% in all cases. (B) The fraction of products for which each transformer provides decisive input on at least one token. Multiple transformers can provide decisive contributions to a given product and a consensus results in no transformer being decisive, so the sum does not equal unity. (C) The reduction in top-*n* accuracy on the testing set upon zeroing out the input to the indicated transformer. (D) Comparison of a R + IR + NMR + MS model trained with missing spectra (blue) with the corresponding fixed input models (green). The cases on the right correspond to the performance with random dropping of one spectral input and supplying a contradictory spectrum (*i.e.*, of starting material or a real product) to one of the spectral transformers. The red bars correspond to the fraction of cases where the contradictory species corresponding to the supplied spectrum was predicted in the top-*n* structures. (E) Three illustrative comparisons of the inferences of different models. (F) The convergence of the accuracy with respect to the number of training data on each of the deduction models.

To test the hypothesis that combining a RtP transformer with one or more StS transformers circumvents the deductive bottleneck in the product identification task, the top-1 and top-5 testing accuracies of the deduction models were compared with the RtP and StS results (Fig. 2A). All the deduction models (even those with fewer spectral inputs) outperform the RtP and StS models by ∼20%, showing a qualitative difference between the inductive and deductive architectures. To clearly illustrate the non-linear impact of combining general reaction knowledge and the spectral information within a single model, we also calculated the top-1 accuracy of a hypothetical RtP + StS model that combines the correct predictions of the two separate models (line in Fig. 2A). Despite this generous accuracy calculation, the best deduction model still outperforms the RtP + StS model by 29%, illustrating the non-additive coupling between the reactant and spectral transformers. The deductive models also show no significant accuracy difference between predicting starting material *versus* expected products. This confirms that the reactant knowledge provided by the RtP transformer also assists with identifying starting material when incorporated within the larger deductive network.

### Evidence for deductive inference

The deductive architecture was motivated by the hypothesis that predictive control might switch between transformers during the token-by-token product decoding. To directly test this, the probability vectors produced by the transformers were individually zeroed out during inference to test whether the most probable overall token predicted by the model changed. If such a swap occurred for at least one token in a product, then the transformer was considered decisive in that decoding (Fig. 2B). The reactant transformer was found to be decisive for at least one token in over 95% of products, followed by the IR transformer at ∼30%. The lower decisiveness of the spectral transformers at least partially reflects their tendency to form a consensus and therefore not be individually decisive. For example, the decisiveness of the IR rises in the R + IR model to 58% and 78% on real and null testing reactions, respectively. Approximately half of the products in the testing set had two or more decisive transformers from the R + IR + NMR + MS model involved in their decoding (Fig. S3†). The mode decoding behavior is to switch between a consensus for the majority of the tokens (60–80%) and one or more decisive predictions for a minority of the tokens (20–40%) (Fig. S4†). This is strong support for the mechanism of dynamic deductive constraints being supplied by the different transformers during the token-by-token inference cycle.

To investigate the overall importance of the different input sources, the accuracy loss upon zeroing out each feature was averaged across the testing data (Fig. 2C). Given the stochastic nature of the decoding, a given input can influence a prediction even if it is not decisive for any particular token. Conversely, even if a transformer is decisive for a particular token, the flexibility of SMILES in decoding the same structure multiple ways means that a correct prediction may still be possible absent that transformer. The accuracy contributions roughly mirror the decisiveness of each transformer (Fig. 2B). In the case of IR, the influence on accuracy is ∼20% larger than the decisiveness measure, whereas for R, NMR, and MS it is marginally smaller. We interpret the relative contributions of the different spectra to reflect the simulation accuracy rather than the intrinsic information content of each spectral source. Nevertheless, there are many cases where even EI-MS makes decisive contributions to top predictions. An extended discussion of decisive behaviors is included in Section 2 of the ESI,† with an additional example showing how different information sources can be decisive for various molecular features (Fig. S5†).

Several additional tests were performed to interrogate the ability of the deductive models to operate in scenarios of incomplete information and even contradictory information (Fig. 2D). For these trials, a version of the R + IR + NMR + MS model was trained from scratch using a ten percent random chance of dropping each spectral input based on the hypothesis that this would reduce the model reliance on consensus formation (see Methods). First, we tested the performance of this model in situations where one or more spectral inputs were unavailable. The performance of the model monotonically decreases on the testing set as spectral information is removed, but the top-1 and top-5 performance remain comparable to the models with fixed inputs (*e.g.*, comparing R + IR + NMR + MS when deprived of IR and NMR data against the R + MS model). The performance remains comparably high in the case where the spectrum being removed is randomized, and for which there is no analog among the fixed input models. These trials show that the deductive architecture is capable of basing predictions on a flexible number of input sources, analogous to the situation in product identification when spectra arrive asynchronously or may be unavailable for a given analyte (*e.g.*, EI-MS may not be available for large molecules).

The R + IR + NMR + MS model trained with missing spectra was also tested in situations with contradictory information by supplying one of the spectral transformers at random with a contradictory spectrum (either starting material or real product) from the others (Fig. 2D, right). The performance in this case is lower than the situation where the model is simply deprived of a spectrum; nevertheless, the model shows the capacity to form a consensus that overrules the predictions of the misinformed transformer. Remarkably, the model still predicts the contradictory species in the top-5 in nearly 40% cases. Although unanticipated, this behavior is more consistent with the supplied evidence than if the model never predicted the contradictory species. This also provides encouraging evidence that this architecture might be extended to predicting product mixtures. For example, a binary mixture of species with large differences in ionization efficiency or oscillator strengths could present similarly to the contradictory use case.

### Illustrative examples and prediction limits

Inspection of some specific testing set examples illustrates the various ways that information is being used by the model (Fig. 2E). The first example shows a case where the IR + NMR + MS StS model fails for a relatively large product molecule, whereas the R + IR + NMR + MS model correctly predicts the product. This improvement reflects the transferable knowledge about organic reactions imparted by the reactant transformer. The second example shows a case where the deduction model fails to predict a product as top-1, but includes it as a top-5 prediction. This example is typical of many of the inaccurate predictions, where the model predicts structural isomers or molecules with similar scaffolds that are difficult to distinguish spectrally. ∼18% of the R + IR + NMR + MS top-1 mispredictions are structural isomers of the target. The third example shows a case where the R + IR + NMR model fails to predict a product as top-5 but the R + IR + NMR + MS model predicts it as a top choice. This case illustrates the complementary information supplied by MS, despite it exhibiting the lowest overall decisiveness and accuracy contribution among the investigated spectra. We judge the low marginal utility of MS to be caused by the relatively poor accuracy of the simulated spectra rather than the intrinsic information content of this spectral source.

A major data curation effort was required to train these models; nevertheless the accuracy *versus* training data size curves for the various models make it clear that there is additional scope for improvement (Fig. 2F). All of the models show clear evidence of saturation that we attribute to two factors. The first is that the performance of the models in identifying real products is already approaching the probable irreducible error of the underlying patent-sourced reaction data (*i.e.*, many of the expected product labels are likely incorrect and cannot be accurately predicted regardless of having more data). The second potential source of saturation is the use of simulated spectra for these models. It is possible that real spectra would exhibit more information and saturate later.

### External case studies

**Fig. 3:**
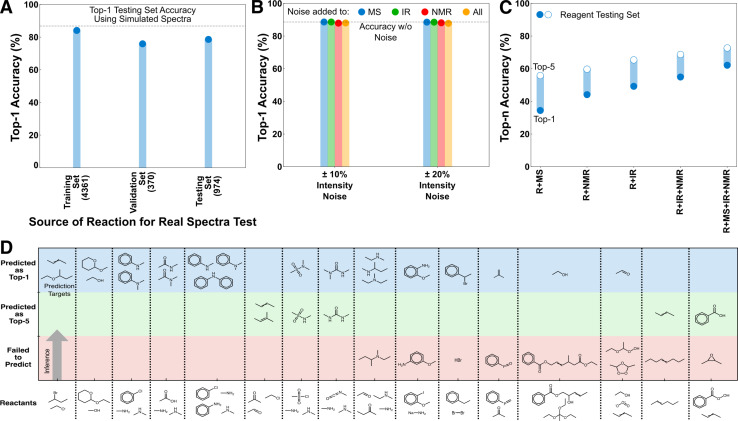
Performance of the deduction models on external testing sets. (A) Performance of the R + MS + IR model in predicting products based on experimental spectra. Experimental IR and EI-MS were sourced from the NIST WebBook for 5705 of the USPTO targets to test model performance on real spectra. The number of samples in each set are shown in parentheses below the label. (B) Performance of the R + MS + IR + NMR model in predicting products based on noised spectra. Inference on the full testing set was reperformed under scenarios with noise on each and all spectral inputs. (C) Comparison of top-*n* performance in identifying reagents that were unseen as prediction targets during training. (D) Performance of the R + IR + NMR + MS model in predicting major and minor products of unseen reactions involving 18 sets of reactants. The products for the 2 sets of reactants that are not shown were not predicted in the top-5 by the model.

Because these models were only trained on predicting starting material and major products using simulated spectra, it was unclear how their performance would translate to predicting the products of side-reactions or other off-target species, or how their performance would translate when using real spectral sources. We curated three external testing datasets, REAL, REAGENT, and MULTI (see Methods) to test the transferability of the model in these scenarios (Fig. 3).

The REAL dataset is made of 5705 reactions from USPTO whose target molecules have both experimental EI-MS and IR spectra (collected from the NIST Chemistry WebBook). As EI-MS and IR are the only provided spectral sources, performance on the REAL dataset was evaluated using a R + IR + MS model trained only using simulated IR and MS spectra. The performance of the R + IR + MS model on the REAL dataset shows a top-1 performance reduction of ∼10% in all scenarios compared with its testing set accuracy when using simulated data (Fig. 3A). No fine-tuning was done to the model, the weights were determined solely from training on simulated spectra. Because all of these predictions use real rather than simulated spectra, they can all be considered an external testing set; however, for clarity we separately present the performance on species that were present in the original training, validation, and testing sets, respectively. With additional fine-tuning the accuracy reduction between the simulated and real predictions could be further reduced. However, we consider this excellent out-of-the-box transferability sufficient to establish that closing this gap is a data challenge rather than an architectural challenge.

A secondary test of transferability to experimental spectra was performed that consisted of adding different noise levels to the simulated spectra. This was done in response to a reviewer suggestion that experimental noise levels might significantly reduce model performance. To test this, we applied noise to the R + IR + NMR + MS model under four scenarios, corresponding to noise applied individually to the spectral inputs or all at once. The noising procedure was as follows: For each non-zero position in the spectra (IR and NMR are discretized in advance), a random choice was selected between increasing/decreasing the peak intensity by a fixed percentage, or keeping the intensity unchanged. Noise levels of 10% and 20% were separately tested, both of which were intended to be relatively high noise levels compared with experimental intensity variability for these analytical techniques. Nevertheless, the top-1 performance of the R + IR + NMR + MS model using the noised inputs are almost identical to original un-noised accuracy in all scenarios (Fig. 3B). This behavior is consistent with the earlier decisiveness testing (Fig. 2B, S3 and S4†) that showed the inference of the mixed-mode models to be based across information sources with the major contribution from the reactant transformer, and thus they are expected to be less sensitive to individual peak intensities of the spectra.

The REAGENT dataset is made of 4952 reactions where the prediction target was a reagent rather than the starting material or expected product, as in the training data (see Methods). Reagent identification was an untrained task for these models and all reagents were unseen as prediction targets during training. The performance trend for reagent prediction is similar to the main testing cases, with a monotonic decrease in accuracy as spectral sources are removed and a baseline accuracy that is above the best StS model (Fig. 3C). The accuracy is still reduced overall, as is expected given the difference between the training task and this task, but nevertheless the transferability to an unseen task is excellent. The RtP model is not compared here because it has ∼0% accuracy on this task, which is a reminder of the qualitative difference between the deductive and inductive architectures despite the high decisiveness of the reactant transformer in the deductive architecture.

The capacity of the models to predict minor products was tested on the MULTI dataset of 18 organic reactants, each with two or more possible products producing a total of 40 distinct reactions, curated from published and textbook sources (see Methods).^22,23^ None of these reactions existed in the training data, and predicting side-products (as opposed to starting material) was not a task that was directly trained for. The R + IR + NMR + MS model can identify the major and minor products in the top-1 for 21/40 of the reactions for 13/18 of the distinct reactants (Fig. 3D, Table S1† has all reactions). Several of the failure cases are also illuminating. For example, the structural isomers of anisidine are largely indistinguishable using the limited analytical sources provided to the model. Nevertheless, the transferability to this unseen task suggests that when provided with additional spectral sources and task-specific training, this architecture is also capable of side-product identification.

## Conclusions

The deductive super-networks studied here were designed to weight evidence from inductive sub-models responsible for digesting individual information sources. This concept was loosely inspired by human deduction, whereby training occurs on specific inductive tasks (*e.g.*, certain types of math, physics, or organic synthesis problems) that are consulted to construct and weight hypotheses and reject solutions in practical scenarios. This idea is also consistent with deductive behavior being an emergent capability of sufficiently expansive inductive subsystems or training datasets. For example, large language models show emergent deductive behavior as evidenced by their ability to respond to non sequiturs, questions that assume certain knowledge, and questions with false premises that contradict established knowledge.^24^ Similarly, the surprising versatility of language models in generative chemical applications and general chemical problem solving has been documented by several groups.^18,25,26^ The initial version of this architecture demonstrated surprising transferability to off-target tasks and in prediction scenarios with partial and even contradictory information. Additional variations on this architecture for product prediction and other deductive problems are immediately possible. Among the most obvious that were left unexplored are finding the optimal manner of combining the inductive sub-models (*e.g.*, more sophisticated couplings beyond the linear reweighting used here) and training the super-network (*e.g.*, training on multiple tasks or contrasting examples).

There are many opportunities for further improving these models and for applications beyond product identification. For example, the current work has not addressed the problem of product identification when the spectra contain product mixtures. Knowledge about the number of species is a powerful deductive constraint that was provided here implicitly through the training data curation; however, this too could be treated as a learnable deduction using an additional classifier or spectral segmentation model to deconvolute spectra for the spectral transformers. This is beyond the current scope, other than to acknowledge the opportunity. Deductive architectures should find application more generally in any prediction scenario where a non-functional one-to-many mapping occurs. These include predictions of materials aging, predictive maintenance, reaction planning, and inverse materials design, among others where missing variables, stochastic factors, or extra degrees of freedom make the prediction problem underdetermined. Such scenarios require deductive reasoning, for which the state-of-the-art is often manual expert analysis of disparate information sources. Deductive ML models of the kind demonstrated here should find use in a multitude of similar applications.

## Methods

### Dataset curation

#### Dataset summary

The product identification dataset curated here consists of 446 330 samples, split between 299 658 samples (249 006 in training, 25 711 in validation and 24 941 in test) corresponding to real product prediction and 146 672 samples corresponding to starting material prediction. Each sample in the dataset is composed of the reactant and reagent SMILES, the simulated EI-MS, IR, and ^1^H-NMR of the prediction target as available features, and the product SMILES as the prediction target. Two versions of the dataset were used, one with reagents distinguished from other reactants using a special token, “>”, and one without. A 80 : 10 : 10 training : validation : testing split was used for all model development. The curation details of this dataset and the data splits are summarized in the remaining sections.

#### Dataset curation

The USPTO reaction dataset originally curated by Daniel Lowe then filtered and split by Jin *et al.* served as the starting point for data curation.^16,17^ This dataset provided reactant : product pairs in the form of SMILES strings that needed to be augmented with spectral data (*i.e.*, EI-MS, IR, and ^1^H-NMR) for each species for use in the product identification learning task. Filtering the reactions for compatibility with the spectral generation workflow (described next) resulted in 299 658 distinct reactions involving 374 681 distinct molecules (counting distinct reactants, reagents, and products).

#### Simulated spectra

Spectra were simulated for all 374 681 distinct molecules in the dataset, because open-source spectral databases are insufficiently large and have limited overlap with the Lowe species to be useful for training a practical product identification model. IR spectra with 4 cm^−1^ resolution from 400–4000 cm^−1^ were generated from the SMILES string of each molecule using the message-passing neural network model published by McGill *et al.*^27^ EI-MS spectra with 1 *m*/*z* resolution from 1 to 999 *m*/*z* were generated using bidirectional neural network model (NEIMS) and rapid approximate subset-based spectra prediction (rassp) model published by Wei *et al.* and Zhu *et al.* respectively.^28,29^ In general, the rassp spectra are more accurate but have size limitations, so NEIMS spectra were used as substitutions wherever rassp spectra were unavailable (about half of the spectra). ^1^H-NMR spectra with 0.0121 ppm resolution from −2 ppm to 10 ppm were generated using Mestrenova v14.3.0.^30^ Spectral generation for both EI-MS and ^1^H-NMR required optimized geometries of each species that were generated using Auto3D.^31^ Reactions from the Jin *et al.* USPTO dataset involving species with more than 30 heavy atoms or elements besides H, B, C, Si, N, P, O, S, Se, F, Cl, Br, and I were discarded to conform to the current constraints of Auto3D.^17^ These exclusions resulted in the final set of 299 658 reactions with real products as prediction targets. Stereochemical tokens were omitted from all training strings to defer a detailed investigation of these prediction behaviors to a future study. There are otherwise no technical obstacles to training these architectures to make stereochemically specific predictions.

#### Null reactions

To test the model's deductive capability, a set of “null reactions” was generated that share the same reactants and reagents as real reactions but with products and input spectra corresponding to one of the reactants. Predicting the product of such reactions corresponds to identifying starting material as an unintended product using the information provided by the spectra. The introduction of null reactions also creates an underdetermined scenario for a RtP model, since a given reactant can yield multiple potential products. Null reactions were generated for each of the 299 658 real reactions. All possible null reactions were generated for reactions with multiple reactants. The USPTO dataset is large enough that some reactants are products of other reactions. In recognition of this, null reactions were discarded if their prediction target matched a real product of any reaction in the dataset. This exclusion was done to avoid accidental information leakage between null reactions and real reactions and also because it yielded a useful 2 : 1 data balance between real and null reactions without further filtering. A total of 146 672 null reactions satisfied this criteria, resulting in a combined dataset of 446 330 reactions (*i.e.*, 146 672 null and 299 658 real) for the product identification task.

#### Dataset splitting

An 80 : 10 : 10 training : validation : testing split was used for model development. The splitting was performed so that all reactions that shared a prediction target were partitioned to the same split. This was done to ensure that the testing and validation sets correspond to unseen prediction targets. For example, if ibuprofen was a product of five different real reactions and two null reactions in the dataset, then all seven would be partitioned to the same split (at random) since they all share the same prediction target (*i.e.*, ibuprofen). This avoids information exchange between tasks, where the model would potentially see the same prediction spectra during training and testing. The total number of real and null reactions, together with their training–validation–test split is summarized in Table 1.

**Table 1:** Dataset split used for deduction model training

	Training set	Validation set	Test set
Real reactions	249 006	25 711	24 941
Null reactions	104 660	12 054	14 810

#### External testing datasets

Three additional datasets, REAL, MULTI, and REAGENT, were curated to test the performance of the deduction models when predicting reactions with experimental spectra, side products, and for identifying reagents as potential products, respectively. The REAL dataset was curated by replacing simulated spectra with experimental spectra collected from NIST Chemistry WebBook. A total 5705 targets (including both null and real targets from USPTO) had spectra available from the WebBook. These targets were split into three groups, based on whether the target species was originally present in the training (4361 reactions), validation (370 reactions), or testing split (974 reactions), respectively. This splitting was done only for the purpose of reporting the results, no fine-tuning was actually done on the R + IR + MS model used for inference in this case study. The MULTI dataset consists of a set of organic reactions with known side-products curated from Grossman's textbook and the dataset compiled by Hartenfeller *et al.*^22,23^ These reactions were combined to produce a total 18 reactants involved in 40 distinct reactions. The REAGENT dataset was curated by identifying all unique reagent species from the main dataset and excluding any that overlapped with targets in the training set or that were incompatible with the spectral generation workflow. This resulted in 3549 distinct reagents. Up to three reactions, if available, from the main dataset involving each reagent was selected at random and the prediction target and input spectra were swapped for the reagent to yield a total 4952 reactions. This dataset tests whether the models are able to identify reagents as a potential isolated product. The spectra of all species in the MULTI and REAGENT datasets were simulated using the same protocol as the main training dataset.

### Neural network architecture

#### Architecture summary

All product identification models used an architecture composed of a reaction transformer, one or more spectral transformers, and a single linear deduction layer. The transformers were adapted from those now typical of neural machine translation (NMT) tasks,^32^ using hyperparameter tuning based on the validation set accuracy. Both reactant and spectral data were pre-processed beforehand and then fed into the attention score calculation module of each transformer through a trainable embedding network. Inference was performed by these models in recursive token-by-token fashion until encountering an end token. An illustration of the R + IR + NMR + MS model architecture is shown in Fig. S1.† The largest model trained here, R + IR + NMR + MS, has ∼30 M weights.

#### Input embedding

The raw reactant input data were represented as SMILES strings, because this is currently the most reliable representation in reaction prediction tasks.^33^ The SMILES strings were tokenized using a standard SMILES vocabulary of 284 possible tokens in addition to a special > symbol used (when present) to separate the reactants and reagents (*e.g.*, solvents or catalysts), a padding token, and special start and end tokens (only present in the decoded product strings). Reactant inputs were converted to fixed 276-length (*d*_seq_) input vectors using padding tokens before being passed to a linear token embedding layer that converted each token to a 256-length vector (*d*_emb_). The dimensions of the reactant input after embedding were [276, 256] (*i.e.*, *d*_seq_ by *d*_emb_). The batch dimension is omitted for clarity from all reported sizes.

The raw simulated ^1^H-NMR, EI-MS, and IR spectra were represented as intensity *versus* ppm, *m*/*z*, and cm^−1^ vectors, respectively. To prepare the ^1^H-NMR and EI-MS spectra for embedding, the intensity values were normalized to a range between 0 and 1, binned by percentile (lower range exclusive, upper range inclusive), then tokenized based on the 100 possible percentile ranges and a special bin for zero (*i.e.*, the percentiles served as a vocabulary for tokenization). The embedding of the IR spectra was identical except that intensities less than 1% were zeroed out to eliminate potential background noise, resulting in 100 total possible tokens rather than 101 (*i.e.*, the zero token for IR includes the first bin in the ^1^H-NMR and EI-MS cases, so there is one less token). The preprocessed input vectors for the IR, ^1^H-NMR, and EI-MS spectra were of length 900 (representing 400–4000 cm^−1^ with a 4 cm^−1^ resolution), 993 (representing −2 ppm to 10 ppm with ∼0.0121 ppm resolution), and 999 (representing 1–999 *m*/*z* with 1 *m*/*z* resolution). The input vectors were then embedded using a linear layer (specific to each transformer but with *d*_emb_ = 256 in all cases) in the same manner as the reactants, resulting in embedded inputs of size [900, 256], [993, 256], and [999, 256] for the IR, ^1^H-NMR, and EI-MS transformers, respectively.

To retain the spatial information of the inputs for use by the models (*i.e.*, token position for the reactants and peak location for the spectra), standard trigonometric positional embedding (*P*) was added to the token-based embeddings according to1
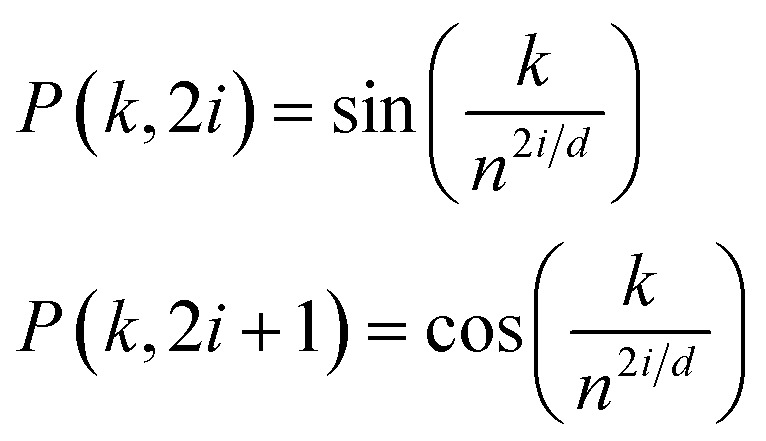
where *k* is the position of the input token, *i* is the position in the embedding dimension, *d* is the hidden dimension (*d*_emb_), and *n* is a convenient constant for determining the relative frequency shift between the sequentially sampled periodic functions (taken to be 10^4^, here).

#### Attention cells

Each transformer is composed of a task-specific encoder and decoder that use two to four attention cells. Each encoder attention cell consists of a sequence of layer norm, multi-head self-attention layer, residual connection, layer norm, feed-forward layer, and residual connection (Fig. S2†). The layer norm is performed before other attention and feed-forward operations with an *ε* value of 10^−6^. Eight attention heads were used, using linear projections of the input embedding dimension to form key and query vectors of length 256 (*d*_k_ = *d*_q_ = 256) and value vectors of length *d*_v_ = *d*_emb_/8 = 32, and the dot-product attention mechanism calculated according to2
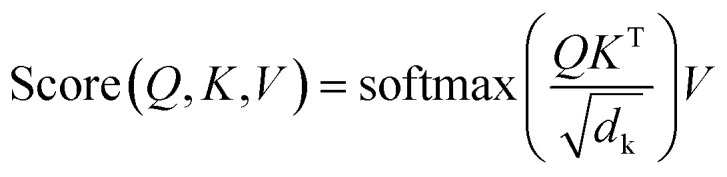
where *Q*, *K*, and *V* are matrices containing the queries, keys, and values for each embedded token (for the first cell, afterwards the derived feature of the previous cell) in the sequence with sizes of [*d*_seq_, *d*_k_], [*d*_seq_, *d*_k_], and [*d*_seq_, *d*_v_], respectively, and 
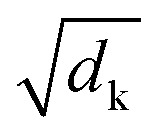
 is a normalization factor. The outputs of each head are catenated along the value dimension to recover a matrix of the same size as the input to the attention layer. The catenated output from the multi-head attention layer is added to the input of the attention cell *via* a residual connection, then passed to a second layer norm and fed to a feed-forward block that consists of a linear layer to project the *d*_emb_-dimension into a 2048-length vector, followed by a ReLU activation layer, and a second linear layer to project the hidden dimension from 2048 back to *d*_emb_. Two drop-out layers with drop-out rate of 0.1 were applied after each linear transformation during training. Finally, the input to the attention cell is mixed with the output *via* another residual connection.

The decoder attention cells used in these models are identical to the encoder attention cells, with the exceptions that the target SMILES embedding is used as an input to the first cell, the multi-head self-attention layer uses masking to restrict non-zero attention calculations to later tokens, and a multi-head cross-attention layer is inserted after the masked multi-head self-attention layer (Fig. S2†). The embedding layer used for the predicted product SMILES is shared across transformers and determined by training. The self-attention masking is identical to that used by Vaswani *et al.*^32^ The multi-head cross-attention layer is identical to the unmasked multi-head self-attention layer in the encoder attention cells, except that the key and value inputs are obtained as linear projections of the embedding dimension of the encoder output and the queries are obtained as linear projections of the embedding dimension of the output of the masked self-attention layer. Layer norms are used before each attention layer and residual connections are used after each attention layer (the same as for the encoder, there is just an extra one of each); all other details (sizes, sequence, number of heads, the final feed-forward layer, *etc.*) are identical to the encoder attention cells.

#### Transformers

All models were constructed from one or more transformers, with each consisting of an encoder, decoder, and terminal linear softmax classifier to predict the next token in the sequence. The encoder and decoder of each transformer were composed of a series of the attention cells described in the previous section. In the case of the reactant transformer, four attention cells were used in the encoder and decoder; whereas, for all spectral transformers only two attention cells were used in the encoder and decoder. A minimal loss in validation accuracy was observed upon reducing the number of attention cells in the spectral transformers and this expedited model training. More transformers might be useful when training on different data sources or other spectral inputs.

The RtP model consists of a single reactant transformer; the various StS models consist of one or more spectral transformers and no reactant transformer; and the various deduction models consist of a reactant transformer and one or more spectral transformers. For each case, the [*d*_seq_, *d*_emb_] output of each transformer is linearly projected along the embedding-dimension to a 288-length vector (*i.e.*, the number of SMILES plus special tokens) with a softmax to predict the probability of the next token.

#### Deductive layer

The models that combine more than one transformer (*i.e.*, the various StS and R + spectra models) are linked together by a single linear layer that projects the 288 × *N* token-probabilities outputted by the *N* individual transformers to predict the next token. Specifically, the outputs of the transformers are catenated to a 288 × *N*-length vector that is linearly projected to a 288-length vector with a softmax to predict the probability of the next token. Because the weights of this linear projection layer are static after training and independent of the input, this layer represents a simple weighting of the evidence from the different transformers that potentially also accounts for any average linear correlations in the token-predictions observed during training.

The linear linkage of the transformers provides two mechanisms by which the task-specific transformers can act as deductive constraints on each other. The first is through the formation of a consensus prediction of the next token. This simple mechanism allows the more confident transformers to potentially overrule one or more less confident transformers in predicting a particular token. The second is through the recursive token-by-token manner in which the product prediction is made. At each step of this process, the prediction string, updated with the token from the last inference, is passed to all transformers to make their individual next-token predictions. This creates a mechanism by which the transformers can perform inference on prediction strings that they never would have encountered *via* a greedy decoding. For example, a particular transformer may be overruled by the others for several tokens, such that it is now performing inference on a partially decoded product scaffold that it would not have predicted on its own. In such a case, the other transformers have acted as a deductive constraint on the transformer.

Other deductive connections are likely useful but have not been significantly explored due to the immediate success of the current architecture for these prediction tasks. The only alternative that was significantly tested was an architecture that terminated in an additive layer rather than a linear projection, which resulted in a marginal reduction in validation set accuracy.

### Training

All models were trained using the Adam optimizer and a batch size of 20. The learning rate, *η*, was linearly increased each update step followed by an exponential decay according to3
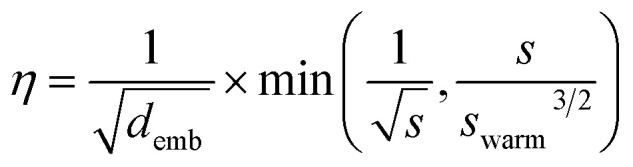
where, *s*, is the step, *s*_warm_ is the number of steps within the warmup phase, and *d*_emb_ is the embedding dimension length. *s*_warm_ was set to 37 500 steps, roughly 4% of the overall training steps, which is consistent with Vaswani *et al.*^32^ No label smoothing was used during training. Early stopping was applied to terminate training if the validation loss did not decrease in the consecutive 30 epochs.

One R + IR + NMR + MS model was trained with random dropping of the spectral sources for use in Fig. 2D of the main text. All other results are for models trained without dropping. For the model trained with dropping, a 10% probability of dropping was separately applied to each input spectrum during training (*i.e.*, on average 1/1000 training samples had no input spectra).

### Inference

During the inference cycle, all models' top-*k* outputs are determined by a beam search with beam size set to five. The beam search algorithm is consistent with the previous implementation published by Schwaller *et al.*^18^ The inference cycle is initiated by feeding the target input with a dummy string only containing the start token “<”. This replaces the target product's SMILES that is used in the training cycle. The model then selects the five most probable tokens decoded from the start string to form five new beams. At each decoding step, each of the beams produces another five candidate strings, and the five candidates with the highest overall probability are selected from the pool of 25 strings, which are then assigned to the new beams for the next decoding step. The decoding of each beam terminates if the end token “$” is predicted as the top-1 or the string length reaches the upper limit of 67.

### Transformer decisiveness and input accuracy reduction

The decisiveness measure was implemented by zeroing out the final probability prediction of each transformer before it was passed to the linear deduction layer. If this caused a change in the top-1 predicted token compared with the unmodified inference, then the transformer was classified as being decisive for that token. According to this definition, one or more transformers can be decisive for a token, and also no transformer can be decisive if a sufficiently strong consensus exists. If a transformer was decisive for at least one token in a given product decoding, then it was classified as being decisive for that product.

The overall accuracy reduction is an alternative measure of input importance that simply reports the reduction in overall top-*n* accuracy when each of the input sources are individually zeroed out. This was implemented by supplying a single padding token to the reactant transformer, and three zero intensity tokens as inputs to the spectral transformers, respectively. The overall accuracy reduction is not necessarily equivalent to the decisiveness of each transformer, because of the flexibility of the SMILES language, which allows the same molecule to be decoded in multiple ways, and the important role of consensus formation in the decoding.

### Ensemble uncertainty estimate

All top-*n* accuracies reported in the main text are from individual models, not ensembles. To provide an estimate of performance uncertainty, five R + IR + NMR + MS models were trained and tested using identical training : validation : testing splits, but with independent weight initializations. Top-*n* accuracy metrics for these models were within 0.5% in all cases.

## Data availability

Figshare repositories have been created for the training, testing, and validation sets (https://figshare.com/articles/dataset/Training_Validation_Test_set_split/25511056), for the model checkpoints (https://figshare.com/articles/dataset/Model_checkpoints/25513519), and for the spectral database (https://figshare.com/articles/dataset/MS_IR_H-NMR_Spectra_Database/25513513). The training scripts and code associated with the multimodal graph + spectrum to graph architecture is maintained on the Savoie group github (https://github.com/Savoie-Research-Group/MultiModalTransformer.git).

## Author contributions

T. J.: conceptualization, investigation, methodology, software, formal analysis, data curation, visualization, writing – original draft. Q. Z.: investigation, methodology, data curation, writing – review & editing. A. B. S.: investigation, data curation, writing – review & editing. B. M. S.: conceptualization, funding acquisition, resources, writing – review & editing, supervision.

## Conflicts of interest

There are no conflicts to declare.

## Supplementary Material

SC-015-D3SC04909D-s001
