# Supplementary material for: Deductive machine learning models for product identification
Source: Chem Sci. 2024 Jul 1;15(30):11995–2005. doi: 10.1039/d3sc04909d (PMC11290435; doi:10.1039/d3sc04909d)
Supplement: SC-015-D3SC04909D-s001 [file SC-015-D3SC04909D-s001.pdf]

Electronic Supplementary Material (ESI) for Chemical Science.
This journal is © The Royal Society of Chemistry 2024

Deductive Machine Learning Models for Product Identification

Tianfan Jin, Qiyuan Zhao, Andrew B. Schofield, and Brett M. Savoie*

Davidson School of Chemical Engineering, Purdue University, West Lafayette, IN, 47906

E-mail: bsavoie@purdue.edu

1 Figures Referenced in the Main Text

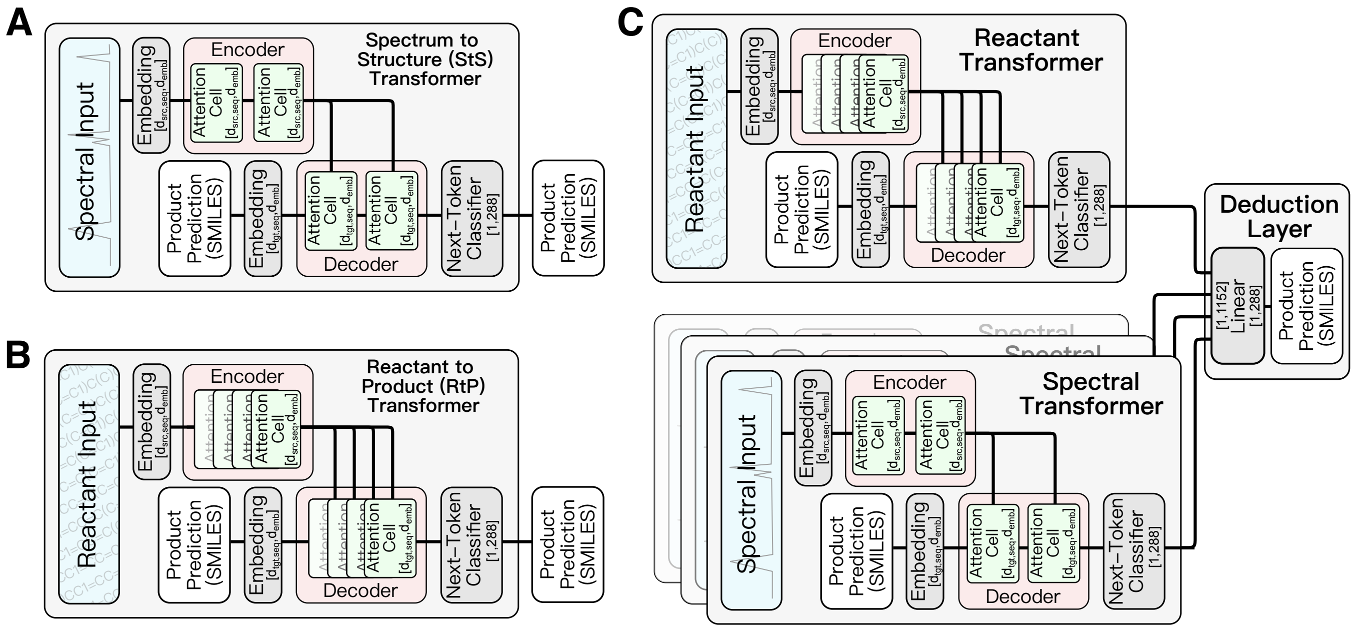

Figure S1: Overview of the different architectures developed in this work. (A) A sample spectrum to structure (StS) transformer. StS models with multiple spectral sources can be compared with the Reactant+Spectra models shown in (C), excluding the reactant transformer. (B) The reactant to product (RtP) model architecture. The use of four attention cells in the reactant encoder and decoder was found to significantly increase accuracy for this input. (C) The R+Spectra deductive architecture. The final linear layer projects to a token probability space using the token probabilities of the individual transformers. The input length $d_{\mathrm{src,seq}}$ varies by input source (src), as described in the main text. The target (tgt) sequence length $d_{\mathrm{tgt,seq}}$ is 64 and shared across models. The batch dimension of each model is omitted for clarity.

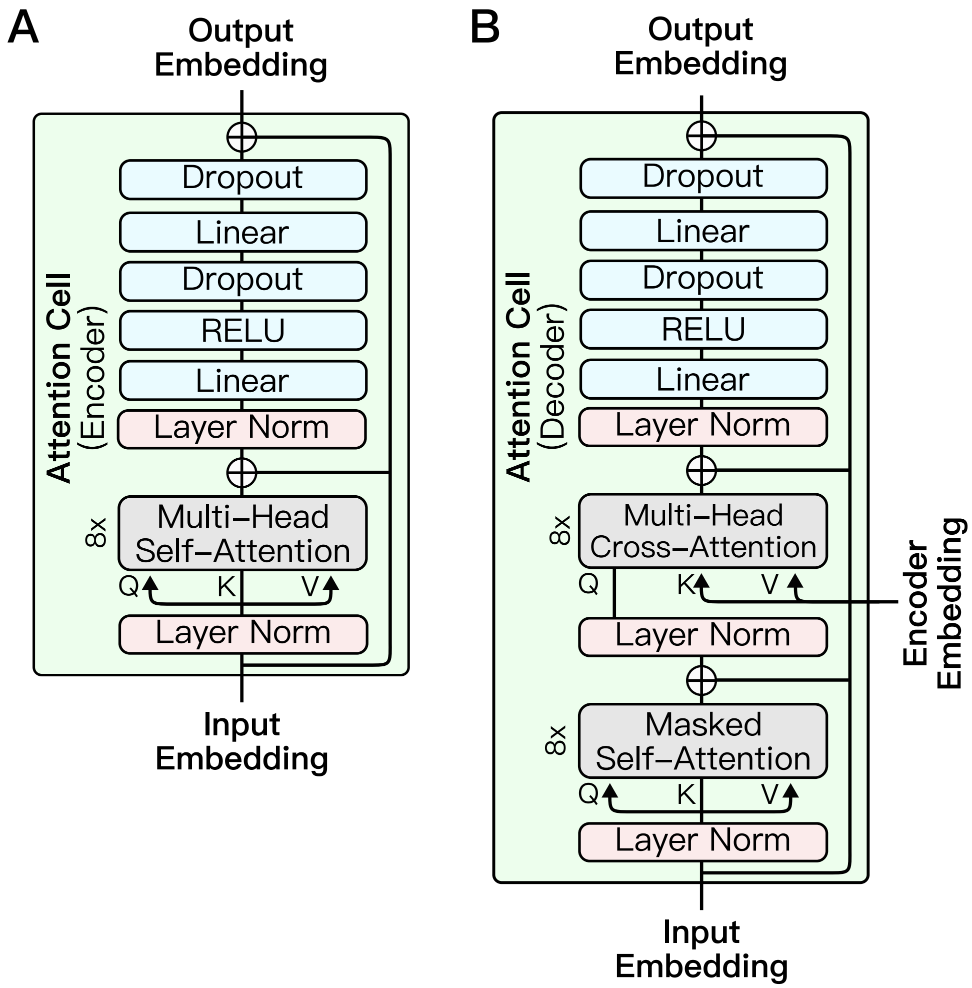

Figure S2: Overview of the attention cells used in the encoder and decoder of each transformer. (a) Encoder attention cell diagram. Each cell consists of typical multi-head attention, layer norm, residual connections, and feed-forward layers. (b) Decoder attention cell diagram. These cells are nearly-identical to the encoder attention cells, except that masking is used for the self-attention layer to limit information exchange to later tokens and there is a cross-attention layer inserted, whose key and value inputs are obtained as linear projections of the embedding dimension of the encoder output and the queries are obtained as linear projections of the embedding dimension of the output of the masked self-attention layer.

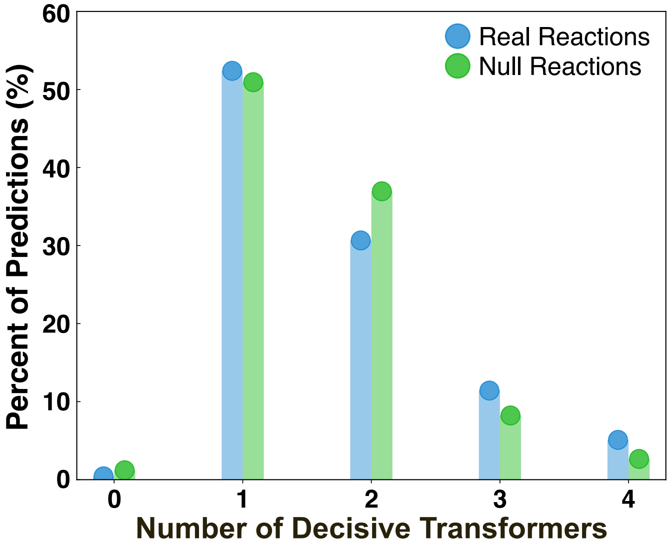

Figure S3: Results from decisiveness testing on the R+IR+NMR+MS model resolved by number of decisive transformers per product. A transformer was considered decisive for a product if it was decisive in decoding at least one token. Multiple transformers can be decisive for a given product, and cases with no decisive transformers mean that all tokens were governed by a consensus.

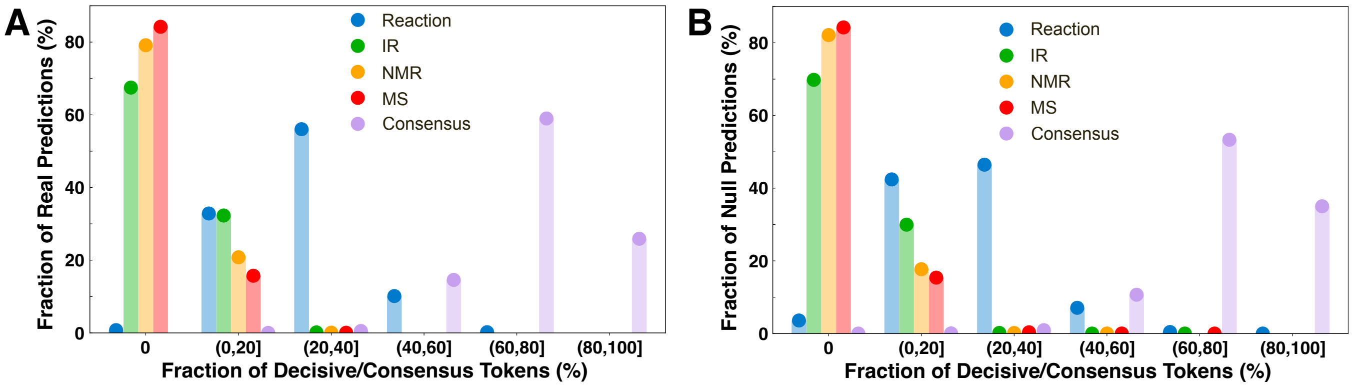

Figure S4: Results from decisiveness testing on the R+IR+NMR+MS model resolved on a per prediction and fraction of tokens basis. (A) Distribution of decisive tokens and consensus tokens across all testing samples corresponding to real product identification. (B) Distribution of decisive tokens and consensus tokens across all testing samples corresponding to starting material identification. A token was classified as a consensus prediction if no individual transformer was decisive in its prediction (i.e., at least two transformers supplied sufficient evidence to overrule the others in the top-1 prediction).

2 Illustrative Inference

We have provided an example to illustrate how the transformers act as deductive constraints upon each other through the recursive graph decomposition that occurs during inference (Fig. S5). The example, 1,4-dibromo-cyclohexane, is a molecule selected at random from the NIST chemistry WebBook and we have used the StS model for the illustration because the reactant-based models show less individual spectral decisiveness. The graph components are shaded based on the different information sources that were decisive in the inference.

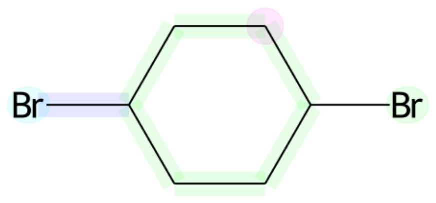

Figure S5: Inference of the MS+IR+NMR model on BrC1CCC(Br)CC1, a structure from the NIST WebBook. Each portion of the graph is shaded by transformer decisiveness: Green (MS decisive), Purple (NMR decisive), Cyan (NMR and MS decisive), Magenta (IR+NMR decisive).

The canonical SMILES for this species is BrC1CCC(Br)CC1. During inference, the starting "Br" is the first token that is decoded and this is almost entirely driven by the MS transformer. The other spectral transformers are not decisive for this token. However, the recursive manner in which inference occurs means that Br is now accepted by all transformers when decoding the rest of the molecular graph. Thus, the presence of the "Br" token is a constraint asserted by the MS that the rest of the transformers condition their inference on. The H-NMR and IR provide

other evidence for inference on the remainder of the graph (e.g., the joint IR/NMR decisiveness for the ring closure).Other notable behaviors include the order of the SMILES inference and the manner in which different spectral sources map to distinct molecular features. Although it is beyond the current scope to analyze in further detail here, we note that the inference patterns often map to typical expert heuristics associated with particular spectral sources. The inference order in this example matched the canonical SMILES, however more generally the model often prefers a non-canonical decoding if it has stronger confidence beginning on another portion of the graph. Similarly, the models often decode distinct SMILES associated with the same molecule in the top-n.

3 Learning Curves

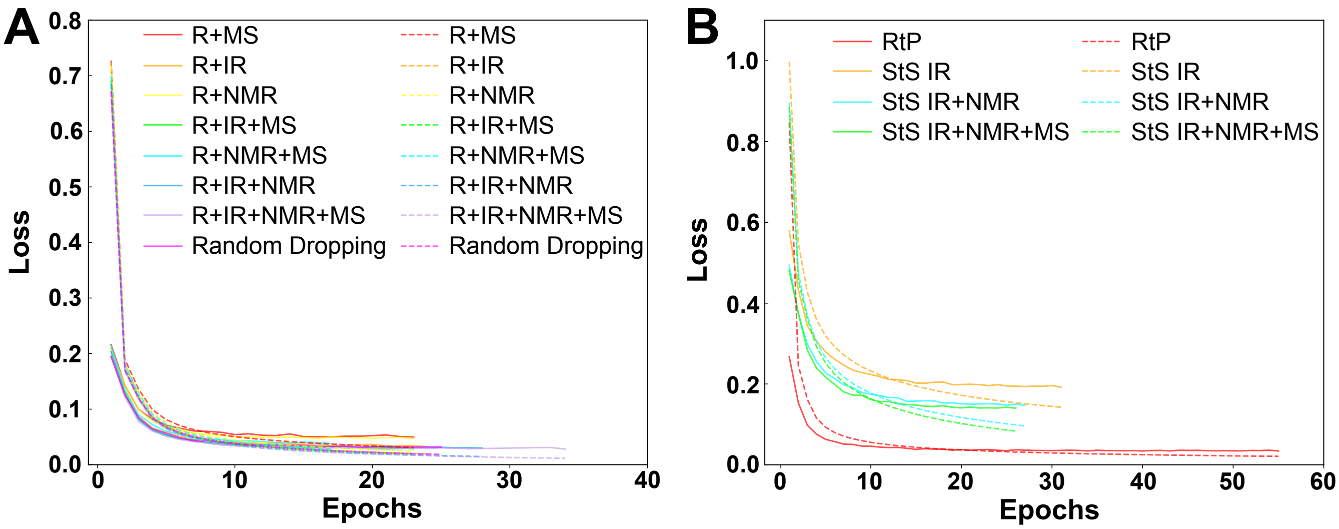

Figure S6: Learning curves for the models trained and tested in this work. Dotted lines correspond to the loss over training data and solid lines correspond to loss over validation data. (A) Composite of the reactant+spectra learning curves. (B) Composite of the StS and RtP learning curves.

4 MULTI Dataset

Table S1: Performance of the R+IR+NMR+MS model in identifying the products of reactions with unintended side-products. None of these examples were present in the USPTO dataset.

Reactants SMILES	Products SMILES	Prediction
C C C (C) Br . C C [O-] >	C C O C (C) C C	Top-1
C C C (C) Br . C C [O-] >	C C = C C	Top-1
O = C 1 C C C C C 1 . [N-] = [N+] = C >	O = C 1 C C C C C C 1	Not in Top-5
O = C 1 C C C C C 1 . [N-] = [N+] = C >	C 1 C C C 2 (C C 1) C O 2	Not in Top-5
C C O C 1 C C C C O 1 . C O >	C O C 1 C C C C O 1	Top-1
C C O C 1 C C C C O 1 . C O >	C C O	Top-1
O = C (O O) c 1 c c c c c 1 . C C = C C >	O = C (O) c 1 c c c c c 1	Top-5
O = C (O O) c 1 c c c c c 1 . C C = C C >	C C 1 O C 1 C	Not in Top-5
C S (= O) (= O) Cl . C N . C N C >	C N S (C) (= O) = O	Top-5
C S (= O) (= O) Cl . C N . C N C >	C N (C) S (C) (= O) = O	Top-1
Cl c 1 c c c c c 1 . C N . C N C >	C N c 1 c c c c c 1	Top-1
Cl c 1 c c c c c 1 . C N . C N C >	C N (C) c 1 c c c c c 1	Top-1
C N = C = O . C N . C N C >	C N C (= O) N (C) C	Top-1
C N = C = O . C N . C N C >	C N C (= O) N C	Top-5
C C = O . C C C (C) = O . C C N C . C N >	C C C (C) N C	Top-1
C C = O . C C C (C) = O . C C N C . C N >	C C N (C) C C	Top-1
C C = O . C C C (C) = O . C C N C . C N >	C C C (C) N (C) C C	Not in Top-5
C C = O . C C C (C) = O . C C N C . C N >	C C N C	Top-1
C C (= O) O . C N . C N C >	C N C (C) = O	Top-1
C C (= O) O . C N . C N C >	C C (= O) N (C) C	Top-1
C O c 1 c c c c c 1 I . N [Na] >	C O c 1 c c c c c 1 N	Top-1
C O c 1 c c c c c 1 I . N [Na] >	C O c 1 c c c c (N) c 1	Not in Top-5
Cl c 1 c c c c c 1 . N c 1 c c c c c 1 . C N . C N C >	C N c 1 c c c c c 1	Top-1
Cl c 1 c c c c c 1 . N c 1 c c c c c 1 . C N . C N C >	C N (C) c 1 c c c c c 1	Top-1
Cl c 1 c c c c c 1 . N c 1 c c c c c 1 . C N . C N C >	c 1 c c c (N c 2 c c c c c 2) c c 1	Top-1
C C C (= O) C C (C) = O . C N N >	C C c 1 c c (C) n n 1 C	Not in Top-5
C C C (= O) C C (C) = O . C N N >	C C c 1 c c (C) n (C) n 1	Not in Top-5
C C (C) = O . C C = O . C C Cl >	C C = C C	Top-5
C C (C) = O . C C = O . C C Cl >	C C = C (C) C	Top-5
C C = C C . [O-] [O+] = O >	C C 1 O O C (C) O 1	Not in Top-5
C C = C C . [O-] [O+] = O >C C O	C C O C (C) O O	Not in Top-5
C C = C C . [O-] [O+] = O >C C O	C C = O	Top-1
C = P c 1 c c c c c 1 . C C (C) = O >	O = P c 1 c c c c c 1	Not in Top-5
C = P c 1 c c c c c 1 . C C (C) = O >	C = C (C) C	Top-1
C C = C C (O) C O C (= O) c 1 c c c c c 1 . C C O C (C) (O C C) O C C >	C C O C (= O) C C (C) C = C C O C (= O) c 1 c c c c c 1	Not in top-5
C C = C C (O) C O C (= O) c 1 c c c c c 1 . C C O C (C) (O C C) O C C >	C C O	Top-1
C C = C C C C >	C C = C C	Top-5
C C = C C C C >	C C C C = C C C C	Not in Top-5
C C c 1 c c c c c 1 . Br Br >	Br	Not in Top-5
C C c 1 c c c c c 1 . Br Br >	C C (Br) c 1 c c c c c 1	Top-1
